# Antidiabetic potential of vanadium complexes combined with olive leaf extracts: a viable approach to reduce metal toxicity

**DOI:** 10.1007/s10534-025-00673-x

**Published:** 2025-02-27

**Authors:** Daniele Sanna, Angela Fadda, Milena Casula, Grazia Palomba, Maria Cristina Sini, Maria Colombino, Carla Rozzo, Giuseppe Palmieri, Carmela Gallo, Dalila Carbone, Laura Siracusa, Luana Pulvirenti, Valeria Ugone

**Affiliations:** 1 Consiglio Nazionale Delle Ricerche, Istituto Di Chimica Biomolecolare, Traversa La Crucca 3, 07100 Sassari, Italy; 2 Consiglio Nazionale Delle Ricerche, Istituto Di Scienze Delle Produzioni Alimentari, Traversa La Crucca 3, 07100 Sassari, Italy; 3 Istituto Di Ricerca Genetica E Biomedica, Consiglio Nazionale Delle Ricerche, Traversa La Crucca 3, 07100 Sassari, Italy; 4 Dipartimento Di Medicina, Chirurgia E Farmacia, Università Di Sassari, Viale San Pietro 43, 07100 Sassari, Italy; 5 Consiglio Nazionale Delle Ricerche, Istituto Di Chimica Biomolecolare, Via Campi Flegrei, 34, 80078 Pozzuoli, NA Italy; 6 Consiglio Nazionale Delle Ricerche, Istituto Di Chimica Biomolecolare, Via Paolo Gaifami, 18, 95126 Catania, Italy

**Keywords:** Vanadium complexes, Olive leaf extracts, Diabetes, Glucose uptake, Intestinal toxicity, GLUT4

## Abstract

Vanadium compounds are known for their antidiabetic properties due to their ability to interfere with numerous mechanisms that lead to the decrease of blood glucose levels. Although some of these compounds have reached clinical trials and have the advantage of being orally administrable, no vanadium-containing drugs are currently available on the market, primarily due to the high doses required, which can lead intestinal and renal problems in case of long-term treatments. In this study, plant extracts obtained from olive leaves (*Olea europaea* L.) were combined with vanadium complexes with established antidiabetic activity with the aim of reducing their metal toxicity and, at the same time, amplifying their hypoglycemic action. The extracts were characterized by chromatographic and spectroscopic methods showing a composition rich in polyphenols and a high antioxidant activity. Formulations containing a vanadium complex (bis(maltolato)oxidovanadium(IV), BMOV, or bis(picolinato)oxidovanadium(IV), BPOV) mixed with different amount of olive leaves extract were tested in vitro to evaluate intestinal toxicity and hypoglycemic activity. The results demonstrated that the plant extracts are generally non-toxic toward human colon fibroblast in the whole range of tested concentrations and some of them are particularly effective in reducing the toxicity of the two vanadium compounds. Further in vitro tests conducted on differentiated human adipocyte cell lines revealed a significant increase in glucose uptake following treatment with the mixed formulations, compared to the effect of the individual components, indicating a synergistic effect. Immunocytochemical assays suggested that the translocation of GLUT4 transporter can be involved in the mechanism of action.

**Supplementary Information:**

The online version contains supplementary material available at 10.1007/s10534-025-00673-x.

## Introduction

Epidemiological data indicate diabetes as one of the most widespread chronic diseases in the world. The adult population suffering from diabetes in 2021 was approximately 537 million and this number is predicted to rise to 783 million by 2045.^1^ To date, the treatment of type 1 diabetes involves insulin-based therapy which has the double disadvantage of subcutaneous administration and the possible onset of insulin resistance phenomena.

In the case of type 2 diabetes, pharmacological therapy is based on the use of different classes of oral drugs capable of reducing blood sugar through different mechanisms of action; unfortunately, prolonged use of commercially available drugs can cause several side effects – such as gastrointestinal issues, weight loss, increased risk of infections, hypoglycemia – and combination therapy is often needed to be tailored to patients’ response (ElSayed et al. 2022). These factors underscore the necessity for innovative approaches in diabetes treatment.

The antidiabetic activity of vanadium compounds has been widely demonstrated in the literature and they present the significant advantage of being suitable for oral administration (Crans et al. 2019). Their ability to reduce blood glucose levels has been attributed to several effects such as stimulation of glucose uptake and metabolism, inhibition of gluconeogenesis and lipolysis (Makinen et al. 2014; Treviño et al. 2019). Since inorganic vanadium salts have a low oral bioavailability (ca. 1–2%), vanadium complexes formed by organic ligands have been widely explored, in particular V^IV^OL_2_, where L is a bidentate monoanionic organic ligand (Thompson et al. 2001; Shechter et al. 2003); these compounds are well tolerated in all animal models of diabetes and more effective than the inorganic salt VOSO_4_ in decreasing blood serum glucose concentration. Among the V^IV^O species with high activity and low toxicity, bis(maltolato)oxidovanadium(IV) and bis(ethylmaltolato)oxidovanadium(IV), designated as BMOV and BEOV respectively, are considered the reference compounds. Maltol and ethylmaltol are U.S. FDA-approved food additives that increase the lipophilicity of the complexes and improve absorption in the gastrointestinal tract. BEOV has reached phase IIa of clinical trials initially with promising results (Thompson et al. 2006; Thompson et al. 2009). Unfortunately, the trial was stopped due to kidney problems observed in some patients and the combination of the pharmaceutical company’s financial problems and patent expiration have made it difficult to finance further clinical trials (Crans et al. 2019). Otherwise, BMOV is currently being developed for other pharmacological applications as Vanadis® drug for the prevention, stoppage, and reparation of secondary tissue injury caused by fire, accidents or heart attack.^2^ Additionally, other clinical trials are currently underway to explore the antidiabetic potential of vanadium compounds. Among these, a microemulsion containing a vanadium salt has successfully completed phase I in the European Union for the treatment of metabolic disorders, such as type 2 diabetes^3^ (Mjos et al. 2014).

In addition to maltol compounds, oxidovanadium(IV) species with N_2_O_2_ coordination appear to be very active in the treatment of insulin-dependent diabetes mellitus (Sakurai et al. 1998); in particular, complexes containing ligands with (N,O) coordination are generally more effective than those with (O,O) coordination (Rehder et al. 2002). Among them, [V^IV^O(pic)_2_(H_2_O)], bis(picolinato)oxidovanadium(IV) or BPOV, has been identified as one of the most promising agents; it was found to be a strong inhibitor of fatty acid mobilization and effective in the treatment of rats with streptozotocin-induced diabetes (Sakurai et al. 1995). Picolinic acid is a catabolite of the amino acid tryptophan, widely used as a food supplement in combination with zinc^4^ and for this reason it is particularly well suited to be used in oral pharmaceutical compositions such as maltol derivates.

Nowadays no V compound is used as antidiabetic drug mainly due to high doses required that can cause gastrointestinal and renal problems in the case of long-term treatments (Crans et al. 2019). Hazardous effects of vanadium compounds have been related to the metal involvement in the generation of reactive oxygen species (ROS), which can lead to cellular damage and toxicity. However, the oxidative stress induced by vanadium-based drugs may also have a beneficial role and it has been associated to their anticancer, antibacterial, and antiparasitic activity (Pisano et al. 2019; Ścibior et al. 2020).

In the context of diabetes therapy, there is the need to develop formulations with a high hypoglycemic action that can allow the administration of lower doses of vanadium to overcome the side effects. Literature data, mostly from animal studies, suggest that dietary antioxidants including ascorbic acid, vitamin E, polyphenols, phytosterols, and extracts from medicinal plants can bring a beneficial effect in vanadium toxicity (Soussi et al. 2006; Marouane et al. 2011; Igado et al. 2018; Koubaa et al. 2019; Zwolak 2020). In particular, the oral co-administration of a tea/vanadate decoction show to provide safe, long-acting hypoglycemic effects in type 1 diabetes mellitus rats while reducing the diarrhetic action of vanadate and organ toxicity (Clark et al. 2004, 2012). The beneficial effect may be attributed to the antioxidant properties of phytocompounds, which help decrease oxidative stress and prevent associated damage. Previous studies have shown that formation of vanadium coordination complexes with polyphenolic ligands can result in a decreased ability to produce ROS (Shukla et al. 2006; Sanna et al. 2016).

Furthermore, it has been demonstrated that using vegetable materials some polyphenols can attenuate intestinal glucose absorption and potentially ‘‘blunt’’ postprandial glucose spikes by inhibition of active uptake via sodium-dependent glucose transporter 1 (SGLT1) and facilitated transport by sodium-independent transporter 2 (GLUT2). Attenuation of glucose transport across the small intestine by polyphenols and their metabolites may be a potential tool of glycemic control (Manzano et al. 2010; Farrell et al. 2013). Therefore, polyphenols, due to their capacity to reduce glucose absorption and to act against the toxic effects of vanadium, could lead to a more effective diabetes treatment when combined with vanadium inorganic ions or vanadium complexes, especially if this combination allows a reduction of vanadium concentration administered.

**Scheme 1:**
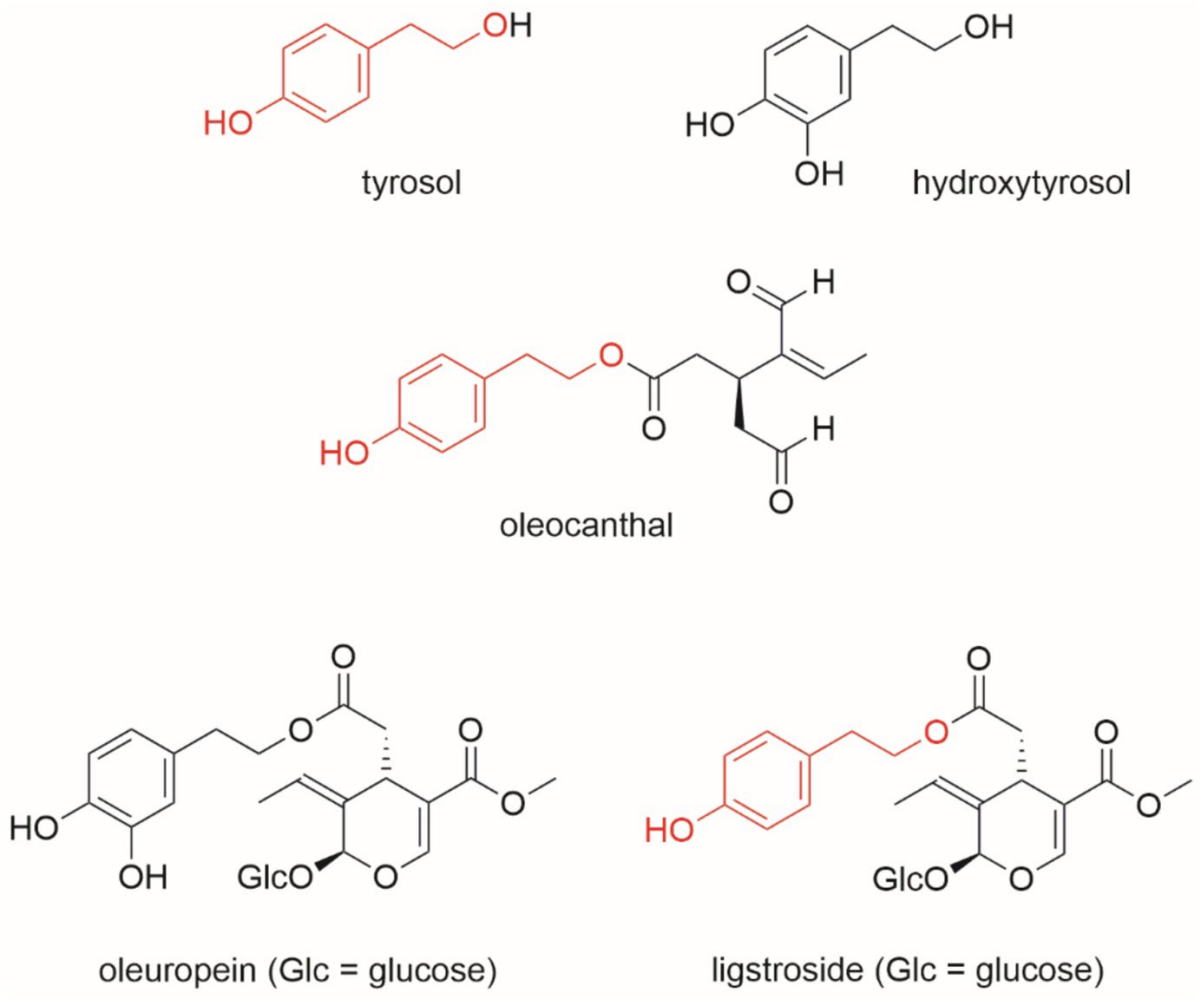
Chemical structures of some of the olive peculiar metabolites object of this work. The common structural features are in red. See Text for details

Decoctions and infusions of olive leaves have been used in traditional medicine as antidiabetic agents (Wainstein et al. 2012; Aidi Wannes et al. 2016; Basuny et al. 2023). The olive tree, one of the most iconic cultures of the Mediterranean area, is characterized by a variegate specialized metabolism, comprising several categories of phenolic compounds such as the simple phenols tyrosol and hydroxytyrosol, hydroxycinnamic acids (chlorogenic acid and verbascoside), flavonoids (mainly flavones apigenin and luteolin) and the secoiridoids ligstroside and oleuropein. Secoiridoids are a mevalonate-shikimate mixed-biosynthesis group of compounds (Koudounas et al. 2015) found exclusively in all the *Oleaceae* plants, including *Olea europaea* L. where they quantitatively dominate the class of bioactive polyphenols (Celano et al. 2019). Olive secoiridoids share the feature of having a tyrosol/hydroxytyrosol moiety in their chemical structure, as evidenced in Scheme 1. These peculiar metabolites are present not only in olive fruits (drupes) and their worldwide-known product olive oil, but also in olive leaves, which are considered the primary and most important waste in olive oil industry (Palmeri et al. 2022).

Oleuropein, tyrosol and hydroxytyrosol, the most important compounds identified in olive leaves, possess antidiabetic properties as demonstrated by in vitro and in vivo studies (Herrero et al. 2011; Talhaoui et al. 2014; Vlavcheski et al. 2019; Acar-Tek et al. 2020).

In this work, new formulations containing vanadium and olive leaf extracts were set with the aim of protecting cells from the toxic effects of vanadium and reducing the doses of metal necessary to obtain the hypoglycemic action. An added value lies in the setup of extraction procedures from waste biomass using solvents that combine environmental sustainability and reduced toxicity with the lipophilicity of the extracts, to facilitate the delivery of phenolic compounds into cells. In particular, ethanol (EtOH), ethyl acetate (EA) and ethyl lactate (EL) were selected as extraction solvents. Ethanol is widely used in the food industry and generally in the preparation of plant extracts and employed as a control in this context. EA and EL are biodegradable, eco-sustainable and safe solvents capable of extracting both apolar (lipids) and polar (polyphenols) compounds. Ethyl lactate derives from biological sources and is commonly used in pharmaceutical preparations and food additives. Ethyl acetate is an organic solvent with low toxicity and is completely biodegradable in a very short time (Aparicio et al. 2009).

**Scheme 2:**
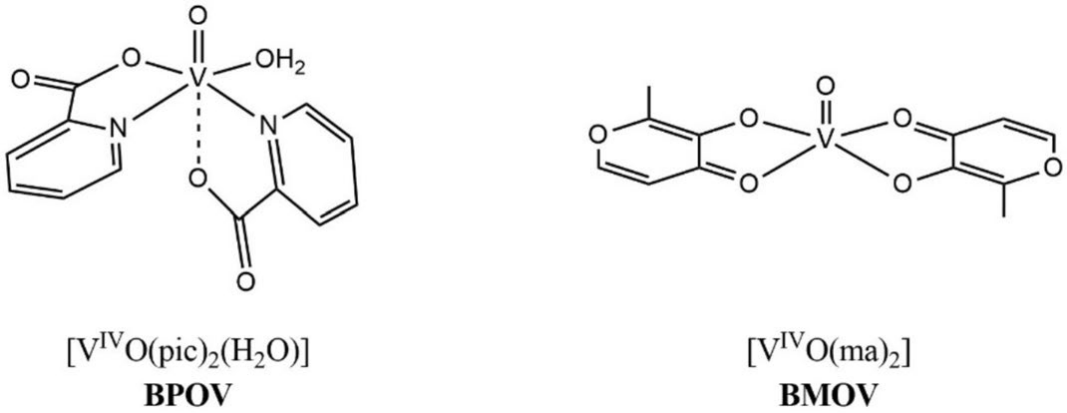
Structures of the complexes BPOV and BMOV

In this study the chemical composition of the extracts and their antioxidant and radical scavenging activity were determined by chromatographic and spectroscopic methods. Formulations containing one vanadium compound, BMOV or BPOV (Scheme 2), and one extract mixed in different ratios were prepared and tested in vitro on human colon fibroblast to assess their intestinal cytotoxicity. The most promising formulations in terms of reduced toxicity were tested on human adipocytes to determine their antidiabetic potential.

## Experimental section

### Materials

All reagents and solvents were of analytical grade unless otherwise specified and used without further purification. 2,2-Diphenyl-1-picryhydrazyl radical (DPPH) was purchased from Alfa Aesar (London, UK), ethanol 96% and ethanol absolute from Carlo Erba (Rodano, MI, Italy). Gallic acid (3,4,5-trihydroxybenzoic acid), quinolinic acid (pyridine-2,3-dicarboxylic acid), ferrous sulfate heptahydrate, hydrogen peroxide (30% w/w), ethanol, ethyl acetate, ethyl lactate, sodium carbonate and Folin-Ciocalteu reagent were purchased from Sigma–Aldrich (Milan, Italy). DMPO (5,5-dimethyl-1-pyrroline N-oxide) was purchased from Enzo Life and used without further purification. Water was purified with a Milli-Q system from Millipore (Millipore Corporation, Billerica, MA, USA).

For chromatographic analyses, HPLC grade water, acetonitrile and methanol were purchased from VWR (Milan, Italy). High purity commercial standards luteolin, luteolin-7-O-glucoside and apigenin-7-O-glucoside were provided by Extrasynthese (Lyon, France), whilst chlorogenic acid, hydroxytyrosol, oleuropein and 3,4-dihydroxyphenilacetic acid (DOPAC) were provided by Sigma-Aldrich (Milan, Italy).

BPOV (bis(maltolate)oxidovanadium(IV)) and BMOV (bis(picolinate)oxidovanadium(IV)) were synthesized according to the methods reported in the literature (Orvig et al. 1995; Lodyga-Chruscinska et al. 2011).

Human colon fibroblast cell line CCD-33Co (CRL-1539™) and preadipocyte cell line isolated from adipose fat tissue hTERT A41hBAT-SVF (CRL-3385™) were purchased from American Type Culture Collection (LGC Standards, Milan, Italy).

DMEM high glucose, fetal bovine serum (FBS), RPMI 1640, Penicillin/Streptomycin, L-glutamine, and 25% Trypsin–EDTA were purchased from Euroclone (Pero, MI, Italy).

Human insulin solution (I9278), dexamethasone (D4902), 3-isobutyl-1-methylxanthine (IBMX, I5879), 3,3',5-triiodo-L-thyronine sodium salt (T3, I6397), indomethacin (I7378), D-pantothenic acid (P5155), biotin (B4639), D-( +)-glucose (G7021), Oil Red O solution (O1391), Formalin solution neutral buffered 10% (HT5012), Bovine Serum Albumin (A-3059), and hematoxylin solution (MHS16) were Sigma-Aldrich products (Milan, Italy).

Rabbit pAb to Glucose Transporter GLUT4 (ab216661), Rabbit specific HRP/AEC (ABC) Detection IHC Kit (ab64260), Aqueous Mounting Medium (ab128982) were Abcam Products (Prodotti Gianni, Milan, Italy).

## Plant material

The olive leaves (*Olea europaea* L. *cv*. Bosana) were collected in spring from a private olive orchard. On the arrival at the laboratory the leaves were inspected to remove damaged ones or leaves with disease symptoms. The selected leaves were washed in tap water and let dry out at room temperature for at least three days. Once dried, the leaves were milled under liquid nitrogen then stored at room temperature and protected from light and moisture until extraction.

## Preparation of the extracts

Three green solvents, ethanol (EtOH), ethyl lactate (EL), and ethyl acetate (EA) were used to prepare the extracts analyzed in this paper. The abbreviation of each solvent is used throughout the text to indicate the respective extracts.

The antioxidant compounds were recovered with a double extraction with 50 mL of solvent. In the first extraction step, 1 g of biomass was stirred with 30 ml of solvent for one hour and centrifuged at 6000 rpm for 20 min. The supernatant was collected, and the residual biomass was re-suspended with the remaining solvent for a further extraction step like the previous one. The solvent was evaporated under vacuum and the dry mass was weighed.

The yield of the extracts was calculated as a percentage by weight of the biomass.

## Characterization of the extracts

### Hyphenated chromatographic analyses (HPLC/Uv–vis-DAD and HPLC/ESI–MS)

Ultra/high performance liquid chromatographic analyses were carried out on an Ultimate 3000 “UHPLC focused” instrument equipped with a binary high pressure pump, a Photodiode Array detector, a Thermostated Column Compartment and an Automated Sample Injector (Thermo Scientific, Milan, Italy). Chromatographic runs were performed on a Gemini C_18_ column (250 × 4.6 mm, 5 μm particle size, Phenomenex, Italy) equipped with a guard column (Gemini C_18_ 4 × 3.0 mm, 5 μm particle size, Phenomenex, Italy). Olive leaves polyphenols were eluted according to the method proposed by Gambacorta et al. 2010. The diode array detector (DAD) was set in the range between 600 and 190 nm, recording the chromatographic profiles at 280, 330 and 350 nm. Collected data were processed through a Chromeleon Chromatography Information Management System v. 6.80. HPLC–ESI–MS analyses were also performed on the same batch of samples using the same conditions (solvents, elution program, guard column, column, injection volume and flow) described above and the same instrumentation described in Palmeri et al. 2017. Quantification of simple phenols and secoiridoids hydroxytyrosol, hydroxytyrosol glucoside, ligstroside, oleuropein and oleuropein aglycone was carried out at 280 nm using calibration curves established with hydroxytyrosol (R^2^ = 0.9997) and oleuropein (R^2^ = 0.9998), whilst DOPAC was quantified at the same wavelength using its corresponding analytical standard (R^2^ = 0.9999). Apigenin glycoside and methyl derivatives were quantified at 330 nm using apigenin 7-O-glucoside (R^2^ = 0.9995); cholorogenic acid (R^2^ = 0.9998) was used at the same wavelength to quantify the corresponding metabolite and caffeic acid. Luteolin, luteolin 7-O-glucoside and their methyl derivatives were quantified at 350 nm using calibration curves established with luteolin (R^2^ = 0.9999) and luteolin-7-O-glucoside (R^2^ = 09994). Analyses were always carried out in triplicate.

### Radical scavenging activity

A solution 1 mM of DPPH in absolute ethanol was prepared. A total of 100 µL of this solution were mixed with a measured volume of variably diluted extract samples in ethanol and then brought to a final volume of 2 mL with absolute ethanol. The samples were kept in the dark at room temperature for 30 min and then the absorbance at 517 nm was measured with a Perkin Elmer Lambda 35 spectrophotometer.

The % of inhibition was calculated as follows:

% of inhibition = (ABS_blank_—ABS_sample_)/ABS_blank_ × 100, where ABS_blank_ is the absorbance of a sample without the extracts. A graph representing the % of inhibition as a function of the extract concentration in the samples was drawn and the experimental points were fitted with a straight-line model. The results of the Radical Scavenging Activity (RSA) are reported as EC_50_ values (expressed as µg/mL).

### Concentration of total phenolic compounds

The concentration of total phenolic compounds was determined with the Folin-Ciocalteu assay according to Fadda et al. 2014. The extracts diluted in water were mixed with the Folin-Ciocalteu reagent (1:1) and with 4 mL of sodium carbonate 7.5%. The reaction mixture was brought to volume (10 mL) and incubated in the dark for 120 min. The absorbance was read at 750 nm with a Perkin-Elmer Lambda 35. The results were expressed as mg of gallic acid equivalents (GAE) per g of extract dry weight (GA calibration curve, concentration range 1–10 mg/L, R^2^ = 0.99).

### Hydroxyl radical scavenging activity

The hydroxyl radical scavenging activity (HRSA) was assayed with the spin trapping method coupled with Electron Paramagnetic Resonance (EPR) spectroscopy. The Fenton reaction, using the Fe(II)-Quin complex as Fe source, was used to produce hydroxyl radicals. The Fe(II)-Quin complex was prepared by mixing FeSO_4_‧7H_2_O and pyridine-2,3-dicarboxylic acid (Quin) to obtain a ligand-to-metal ratio of 5/1 and a Fe(II) concentration of 0.1 mM as described in Sanna et al. 2022. The hydroxyl radicals were trapped with the nitrone spin trap DMPO (5,5-dimethyl-1-pyrroline N-oxide).

Spin trapping experiments with DMPO were carried out at room temperature with a Bruker EMX spectrometer operating at the X band (9.4 GHz) equipped with a HP 53150 frequency counter using a Bruker AquaX capillary cell. Results are expressed as gallic acid equivalents (GAE) reported as mmoles GA g^−1^ d.w., based on a calibration curve (R^2^ = 0.99, gallic acid 80–200 mM). The EPR instrument was set under the following conditions: modulation frequency 100 kHz; modulation amplitude 1 G; receiver gain 1 × 10^5^, microwave power 20 mW.

## Colon fibroblast cell culture and cytotoxicity assay

The human colon fibroblast cell line CCD-33Co was cultured in T75 flasks in a humidified atmosphere with 5% CO₂ at 37 °C using Fibroblast Growth Medium (Promocell C-23010) enriched with Supplement mix containing only Fibroblast Growth Factor and Insulin according to manufacturer instructions. Cells were cultured with 5% FBS without antibiotics and cytotoxic assays were performed without serum.

The cytotoxic effects of vanadium compounds (BPOV and BMOV), the individual extracts (EtOH, EL and EA), and all their combinations were evaluated using the sulforhodamine B assay (Gallo et al. 2020) 24 h after treatment with the test solutions prepared as described below.

The dried extracts were initially suspended in the minimum amount of DMSO (stock solutions stored at − 80 °C) and then diluted in the appropriate culture medium. The toxicity tests of the extracts were performed over a wide concentration range (10–319 µg/mL EL, 10–265 µg/mL EtOH, 10–231 µg/mL EA), considering the solubility limit of the extracts and a DMSO amount less than 0.1% in the final solutions.

The solid BMOV and BPOV compounds were dissolved directly in the culture medium and tested over a wide concentration range (7.5–2000 µM).

To evaluate the protective effect of the extracts on the cytotoxicity of vanadium, mixtures containing a fixed concentration of BMOV or BPOV and increasing concentrations of extract were prepared. The concentrations of BMOV/BPOV were chosen based on the cytotoxicity values of the individual compounds; specifically, a vanadium concentration that has a cytotoxic effect of about 40% cell death compared to untreated controls was selected (BPOV 2 mM, BMOV 0.5 mM).

Before treating the cells with the test compounds, all solutions were filtered through 0.22 µm cellulose acetate disk filters to ensure sterility.

## Preadipocyte cell culture and differentiation

The human preadipocyte cell line hTERT A41hBAT-SVF was cultured in T75 flasks using DMEM/H medium supplemented with 10% fetal bovine serum (FBS), 1% penicillin/streptomycin, and 1% L-glutamine in a humidified atmosphere with 5% CO₂ at 37 °C. The medium was changed every 2–3 days. At 60–80% confluence, the cells were treated with 0.25% Trypsin–EDTA and seeded into 96-well plates at an initial density of 2000 cells/well. The cells were grown in complete DMEM/H for 5–6 days until confluence was reached and then incubated for 14–20 days in a differentiation medium composed of DMEM/H containing 5 mM IBMX, 0.1 µM dexamethasone, 0.5 µM human insulin, 2 nM T3, 30 µM indomethacin, 17 µM pantothenic acid, 33 µM biotin, and 2% FBS (Xue et al. 2015). The differentiation medium was replaced every 2–3 days until differentiation was achieved.

## Lipid staining

Adipocyte differentiation was assessed by Oil Red O staining to visualize lipid accumulation within the cells. The cells were washed with PBS, fixed in 10% formalin for 1–2 h, and treated for 1 h in the dark at room temperature with a 0.5% Oil Red O solution in isopropanol filtered and diluted with distilled water in a 3:2 ratio. After washing with distilled water, the cells were observed under a light microscope.

## Glucose uptake assay

Differentiated adipocytes in 96-well plates were initially subjected to serum starvation for 3 h in RPMI containing 400 mg/dL glucose and 1% BSA (bovine serum albumin). After removing the medium, the cells were treated with solutions containing the test compounds dissolved in RPMI (400 mg/dL glucose, 1% BSA) at different concentrations.

The solutions containing BPOV, extracts (EL and EtOH), and their mixtures were prepared as described in Sect. "Colon fibroblast cell culture and cytotoxicity assay". The vanadium/extract mixtures were prepared using a fixed BPOV concentration of 63 µM.

The remaining glucose concentration in the medium after treatment with the test formulations was measured using a specific portable system for cell culture media (GlucCell® glucose monitoring system). The percentage of glucose consumed after 48 h of treatment was calculated relative to the basal glucose consumption measured on untreated cells (negative control). Human insulin was used as a positive control. All the experiments were performed in triplicate and repeated at least three times.

## GLUT4 immunocytochemistry assay

Immunocytochemical staining was performed using the standard avidin–biotin complex (ABC) technique with a rabbit-specific HRP/AEC Detection IHC Kit. Undifferentiated preadipocytes were seeded in duplicate chambers of an eight-chamber glass slide (Nunc® Lab-Tek® II Chamber Slide™) and induced to differentiate using the previously described differentiation medium. Differentiated adipocytes were subsequently treated with BPOV, EL and EtOH extracts, and their combinations as outlined in Sect. "Preadipocyte cell culture and differentiation".

Following 24 h of treatment, cells were fixed with 10% formalin for 20 min, washed with PBS, and incubated at room temperature for 1 h with a diluted rabbit polyclonal GLUT4 antibody.

After PBS washes, a biotin-conjugated secondary antibody was applied for 10 min, followed by streptavidin-HRP incubation for 10 min at room temperature. Staining was developed using 3-amino-9-ethylcarbazole (AEC) substrate chromogen. Finally, slides were rinsed with distilled water, counterstained with Mayer’s hematoxylin, washed in running tap water, and mounted with a mounting medium. Analysis was performed on an Olympus BX61 microscope, and images were captured at 20 × magnification using Optika Vision Lite Software 2.1.

## Statistical analysis

Analysis of variance was performed with GraphPad Prism8 for windows software (GraphPad Software inc.la Lolla, CA, USA). The data of extracts yield, total phenolic compounds concentration, and radical scavenging activity were analyzed according to a single factor (one-way ANOVA) randomized complete block design and the mean comparisons were calculated by Tukey’s test, *P* ≤ 0.05. Glucose uptake data were analyzed according to a single factor (one-way ANOVA) and mean comparisons were calculated by Dunnet’s test,* P* ≤ 0.05. Statistical significance of cytotoxicity tests was established by two-way ANOVA and Sidak’s test was used for multiple comparisons.

## Result and discussion

### Total phenolic compounds and antioxidant activity

In this work, three different solvents were used for the extraction of phenolic compounds from olive leaves, namely ethyl lactate, ethyl acetate, and ethanol. Ethanol and ethyl lactate have a similar extraction efficiency, while that of ethyl acetate was significantly lower (Table 1). However, despite the comparable extraction yields of ethanol and ethyl lactate, the total phenolic content of the ethanol extracts was twice that of the ethyl lactate extracts. Ethyl acetate, although giving a much lower extraction efficiency than the other solvents, extracted an intermediate amount of total phenolic compounds.

**Table 1:** Extract yield, concentration of total phenolic compounds and antioxidant activity parameters of the extracts

Extraction solvent	Extract Yield % from biomass (w/w)*	Total polyphenols (FC test) (mg GAE/g d.w.)*	Radical scavenging activity (EC_50_ µg/mL)*	Hydroxyl radical scavenging activity (µmoles GAE/g d.w.)
Ethanol	28.59 ± 2.47 a	78.65 ± 9.67 a	63.59 ± 9.33 b	6.01 ± 1.58
Ethyl Lactate	27.90 ± 3.98 a	38.93 ± 5.22 b	152.12 ± 12.20 a	–
Ethyl Acetate	8.45 ± 0.27 b	51.55 ± 0.95 b	62.54 ± 3.28 b	–

^*^In each column grouping, means separation was performed by Tukey’s test (*P* ≤ 0.05)

The radical scavenging activity of the three extracts is consistent with the polyphenolic compounds content. The EC_50_ value in ethanol is ca. one half in comparison with that in ethyl lactate indicating a higher antioxidant activity of the former. Hydroxyl radical scavenging activity was measurable only in the ethanol extract, because the ethyl lactate and ethyl acetate extracts, after solvent evaporation, were insoluble in water.

## Characterization of olive leaf extracts

As already stated, the secondary metabolism of olive leaves include several classes of phenolic compounds: simple phenols, hydroxycinnamic acids, flavonoids and secoiridoids, the last class being considered as a “signature” of *Olea europaea* L. Being specialized metabolites, the content of these molecules in olive leaves depends on genetic and environmental factors (Siracusa et al. 2014) such as cultivar, bearing cycle, and seasonal variations. The compositional features of the corresponding extracts from this matrix vary depending on the solvent/mixture of solvents utilized for the extraction; as example, it was previously demonstrated that secoiridoids and particularly oleuropein are better extracted from olive leaves by using distilled water rather than alcohols or hydroalcoholic mixtures (Monteleone et al. 2021). A representative HPLC chromatogram corresponding to the ethyl lactate extract from olive leaves obtained in this work is depicted in Fig. S1. Sixteen chromatographic signals were tentatively identified by comparing their retention times and spectral data (UV–Vis and MS) with those previously studied in our group (Monteleone et al. 2021; Palmeri et al. 2022); as expected, all the extracts contain several simple phenols (peak 1, hydroxytyrosol-glucoside; peak 2, hydroxytyrosol; peak 3, 3,4-dihydroxyphenilacetic acid), numerous apigenin derivatives (peaks 6, 9, 10, 12) as well as luteolin derivatives (peaks 7, 8, 13) including luteolin itself (peak 16). A considerable number of flavone methyl-derivatives were detected (peaks 9, 10, 12, 13); the presence of these compounds is likely to depend on the extraction system used, in agreement to what already reported (Luca et al. 2024). Quantitatively speaking (see Table 2), oleuropein is undoubtedly the main compound regardless the extraction medium utilized; its amount peaks in the EtOH extract (2.25 mg/100 mg) followed by EL extract (1.87 mg/100 mg) and finally EA extract (1.06 mg/100 mg). Generally, ethyl acetate seems not the right choice for a high-yield extraction of olive polyphenols; on the contrary, ethyl lactate represents a good compromise between lipophilicity and extraction strength for this matrix (3.27 mg total polyphenols/100 mg) especially when compared with ethanol (3.63 mg total polyphenols/100 mg extract) (Fig. S2).

**Table 2:** Individual and total specialized metabolite amounts, reported in mg/100 mg extract, in extracts from olive leaves obtained with different solvents

peak	compound (mg/100 mg)	EL	EA	EtOH
1	hydroxytyrosol hexoside	0.0717	0.0074	0.0550
2	hydroxytyrosol*	0.1430	0.0131	0.0748
3	3,4-dihydroxyphenilacetic acid (DOPAC)*	0.0237	0.0015	0.0045
4	chlorogenic acid*	0.0138	0.0004	0.0232
5	caffeic acid	0.0021	0.0022	0.0075
6	apigenin hexoside derivative	0.0344	0.0018	0.0545
7	luteolin di-O-hexoside	0.0646	0.0061	0.0669
8	luteolin hexoside—pentoside	0.3756	0.0610	0.5201
9	methylapigenin di-O-hexoside	0.0203	n.d	0.0260
10	methylapigenin hexoside-deoxyhexoside	0.0746	0.0239	0.0943
11	oleuropein*	1.8751	1.0592	2.2506
12	methoxyapigenin hexoside	0.2278	0.0420	0.3804
13	methoxyluteolin hexoside	0.0416	n.d	n.d
14	ligstroside	0.1492	n.d	n.d
15	oleuropein aglycon	0.1050	0.0487	n.d
16	luteolin*	0.0488	0.0185	0.0754
	simple phenols	0.2385	0.0219	0.1343
	hydroxycinnamic acids	0.0159	0.0026	0.0307
	flavones	0.8876	0.1534	1.2177
	secoiridoids	2.1293	1.1079	2.2506
	***total polyphenols***	***3.2713***	***1.2858***	***3.6333***

^*^Quantified using the corresponding commercially available analytical standard; results are reported as mean of three replicates. See Experimental section and text for further details

## Cytotoxicity assay

The cytotoxicity of vanadium compounds, plant extracts and their combination towards cellular models of the human intestine (CCD-33Co cell line) was determined by the SRB assay.

The results show that the cytotoxicity of BMOV and BPOV is low on the tested cell line, with IC_50_ values higher than 0.5 mM.

**Fig. 1:**
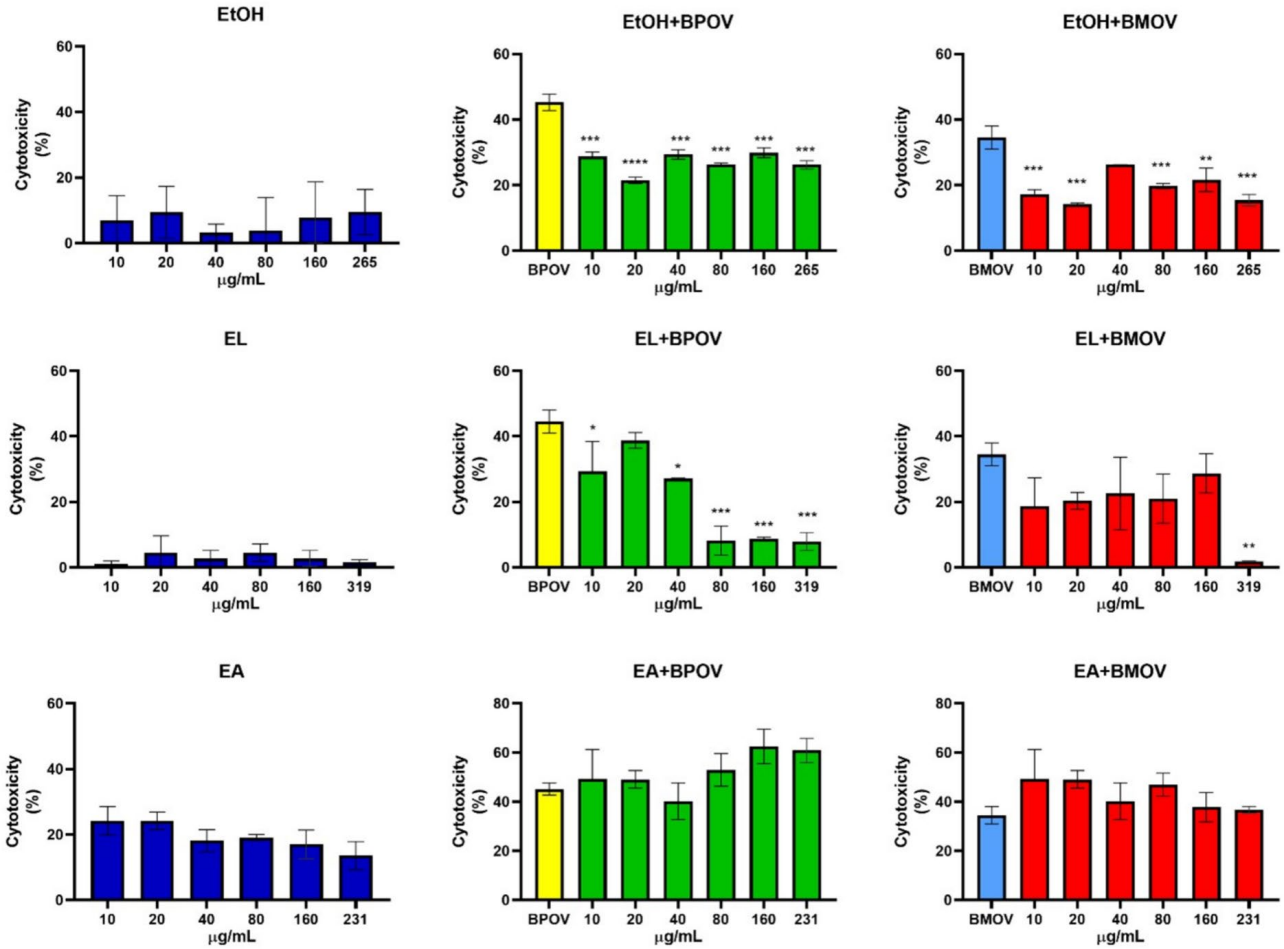
SRB assay conducted on CCD-33Co cells at 24 h of incubation. Data are reported as percentage of cytotoxicity (%). Blue graphs show cytotoxicity of EtOH, EL, EA extracts in the range of concentrations from 10 to 319 µg/mL. Green graphs report cytotoxicity of the combination of EtOH, EL, EA with BPOV compared to the treatment of only BPOV 2 mM (BPOV, yellow) and red graphs reports cytotoxicity of the combination of EtOH, EL, EA with BMOV compared to the treatment of only BMOV 0.5 mM (BMOV, light blue) at the concentrations reported above. Statistical significance represents the significant difference between vanadium with the mix of the tested extracts and vanadium alone considered as a control (*P < 0.05, **P < 0.01, ***P < 0.001, ****P < 0.0001) was established by two-way ANOVA. Sidak’s test was used for multiple comparisons

The plant extracts were found to be substantially non-toxic in the entire range of concentrations tested (10–319 µg/mL) without showing the dose–response trend typical of cytotoxic substances; in particular, cell death percentages remained below 10% for EtOH and EL, and below 25% for EA (Fig. 1, blue graphs).

EtOH and EL extracts can significantly reduce the toxicity of the two vanadium compounds in the entire concentration range and some formulations were found to be particularly effective (i.e. the combination of EL with BPOV is able to almost completely cancel the toxic effect of the reference vanadium compound).

EA extracts, instead, do not show any beneficial effects when combined with BPOV or BMOV giving cytotoxicity values close to those of the reference metal compounds or slightly higher (Fig. 1, green and red graphs).

## In-vitro antidiabetic activity

The hypoglycemic effects of the formulations have been determined by measuring the glucose consumption on adipocytes treated with both the individual compounds and the V/extracts combinations in presence of a fixed amount of glucose in the medium. In particular, EL and EtOH extracts and their combinations with BPOV have been taken into account considering the promising results obtained by the cytotoxicity assays.

**Fig. 2:**
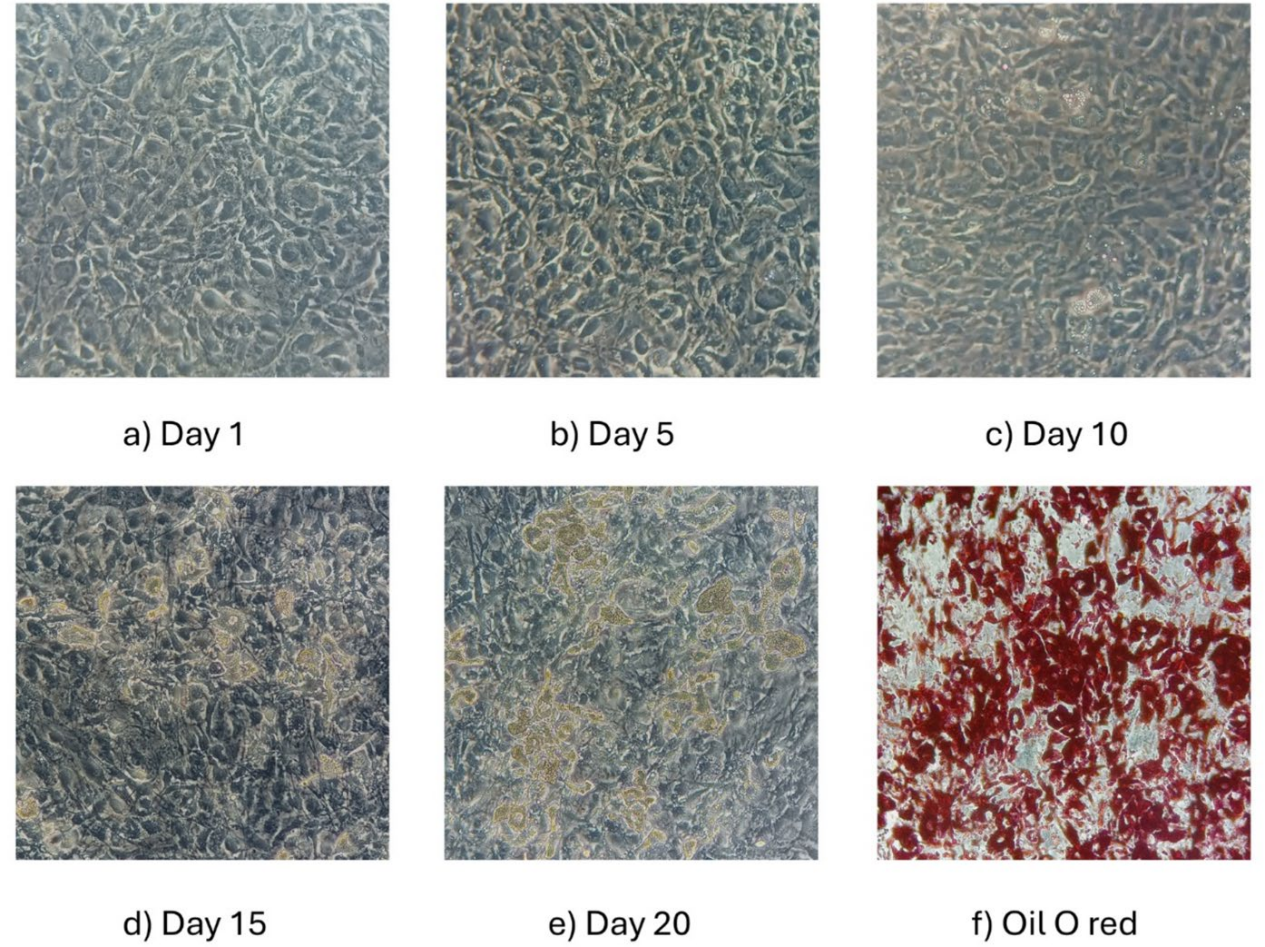
Differentiation process of human pre-adipocytes (**a–e**, day 1–20) and Oil O red staining of differentiated adipocytes (**f**). Inverted microscope images at 20 × magnification

The cells were initially grown and differentiated in 96-well plates as described in the experimental section. The differentiation to mature adipocytes was confirmed by Oil red O staining which allows to mark lipid droplet accumulation (see Fig. 2).

Preliminary tests allowed to identify the minimum concentration of vanadium capable of stimulating glucose absorption in human adipocytes (63 µM) which was used to prepare the formulations with EL and EtOH.

**Fig. 3:**
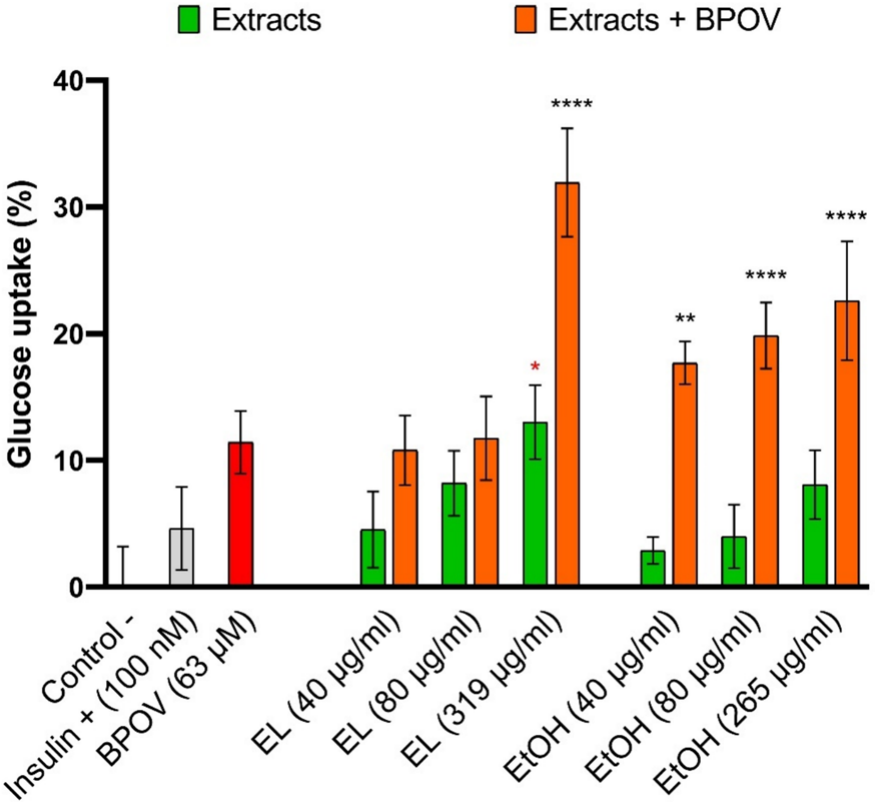
Glucose uptake (%) in human adipocytes treated with insulin (positive control), BPOV, EL extracts, EtOH extracts, and mixtures EL + BPOV and EtOH + BPOV. Untreated cells were taken as negative control. Statistical significance (*P < 0.05, **P < 0.01, ***P < 0.001, ****P < 0.0001) was established by one-way ANOVA. Mean comparisons were calculated by Dunnet’s test, *P* ≤ 0.05. In red statistical significance *vs* insulin (positive control); in black statistical significance *vs* BPOV

The results obtained are summarized in Fig. 3 (where the percentage of glucose consumption refers to the negative control, i.e. untreated cells, basal consumption). It can be noticed that BPOV itself is effective in improving glucose absorption and its effect is even greater than that of insulin (Fig. 3, red column). As regards the extracts administered individually, both EtOH and EL showed a discrete concentration-dependent hypoglycemic action (Fig. 3, green columns), while in presence of BPOV a significant increase in glucose absorption was observed (Fig. 3, orange columns). In particular, the combination of BPOV with EL at the highest concentration appears to be the most effective with a significant increase in the glucose uptake compared to that of the individual components.

Considering the promising effects of the new mixed formulations on decreasing glucose concentration in the extracellular medium, further aspects concerning their mechanism of action were investigated.

In a healthy state, glucose homeostasis is maintained by insulin which promotes glucose uptake into the muscle and adipose tissues and suppression of glucose production in the liver. Adipocytes, hepatocytes, striated muscle cells, cardiac and skeletal cells, all have membrane insulin receptors which initiate a signaling cascade culminating in the translocation and fusion of glucose transporter GLUT4 intracellular vesicles to the cell membrane, greatly increasing the diffusion of glucose to the cell, and in the inhibition of catabolic processes and promotion of anabolic ones. In the diabetic state, which can be caused by an insulin deficiency (type-1 diabetes) or insulin resistance (type-2 diabetes), the utilization of GLUT4 is downregulated leading to the increase in blood glucose concentration.

Previous studies suggested that oxidovanadium(IV) compounds can exert the hypoglycemic effect by activating the insulin signaling pathway, leading to the GLUT4 translocation from intracellular vesicles to the plasma membrane (Mohammad et al. 2002; Basuki et al. 2006; Hiromura et al. 2007).

**Fig. 4:**
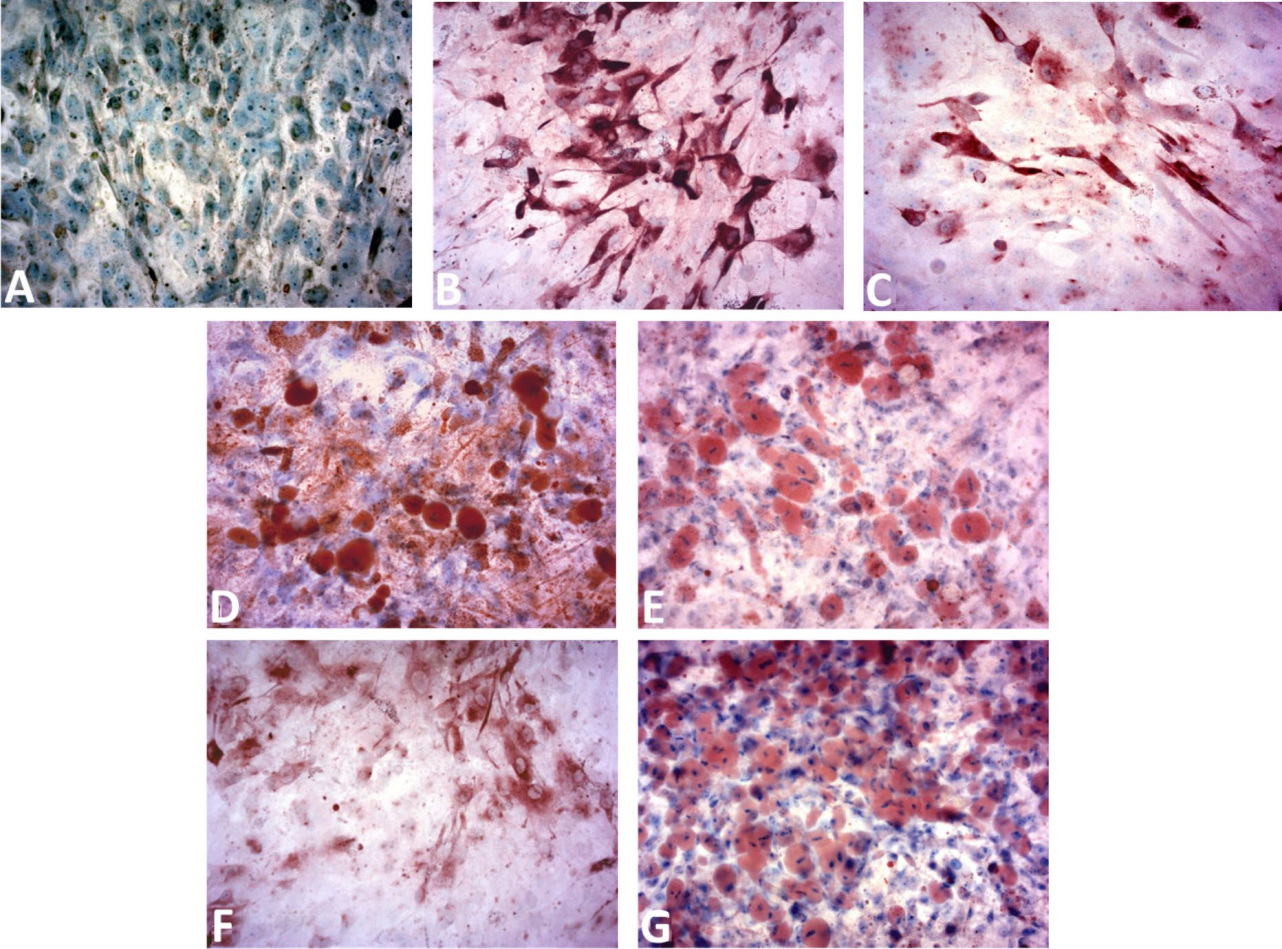
Immunocytochemical staining of differentiated adipocytes subjected to various treatments for GLUT4 (20 × magnification)**.** Red-pink staining indicates positive GLUT4 expression localized exclusively in the cytoplasm (3-amino-9-ethylcarbazole), while blue/violet staining shows nuclei counterstained with hematoxylin. The images represent **A** Untreated adipocytes; **B** Adipocytes treated with BPOV 63 µM; **C** Adipocytes treated with insulin 100 nM; **D** Adipocytes treated with extract EL 80 µg/ml; **E** Adipocytes treated with extract EL + BPOV (EL 80 µg/ml, BPOV 63 µM); **F** Adipocytes treated with extract EtOH 80 µg/ml; **G** Adipocytes treated with extract EtOH + BPOV (EtOH 80 µg/ml, BPOV 63 µM)

In the present study, the expression of GLUT4 in human adipocytes treated with V/extracts formulations and the single components was evaluated by ABC immunocytochemistry to assess if their hypoglycemic action is correlated to this mechanism of action (Fig. 4). Adipocytes treated with either insulin (Fig. 4C) or BPOV (Fig. 4B) showed an increase in the expression of GLUT4 compared to controls (untreated cells, Fig. 4A) confirming the activation of the cascade mechanism that determines the translocation of GLUT4 towards the cytoplasmic membrane and the increase in glucose uptake. The same effect was observed for the V/extracts mixtures (Fig. 4E, G) and the extract EL (Fig. 4D) in less extent for the EtOH extracts administered individually (Fig. 4F).

## Conclusions

This study highlights the potential of vanadium compounds in combination with olive leaf extracts for obtaining novel and safe formulations in antidiabetic therapy. Vanadium compounds are known for their insulin-mimetic effects, and various strategies have been proposed to mitigate the side effects associated with the high doses necessary for their therapeutic efficacy. A promising approach involves combining vanadium compounds with plant extracts possessing antioxidant and hypoglycemic properties; this combination may enhance pharmacological effectiveness of metal complexes at the same time helping to reduce the intrinsic metal toxicity which can arise from their ability to promote oxidative stress.

In this work, the extracts were obtained by sustainable methods using biodegradable solvents such as ethanol, ethyl acetate and ethyl lactate and waste biomass as vegetable matrix. Among the solvents tested, EtOH proved to be the most effective for extracting phenolic compounds from olive leaves, yielding extracts with the highest antioxidant capacity and phenolic content. EL showed comparable extraction efficiency, while EA demonstrated lower effectiveness in both phenolic yield and antioxidant activity. The chromatographic and spectroscopic characterization confirmed the presence of bioactive compounds such as the simple phenol hydroxytyrosol and the secoiridoid oleuropein together with several flavonoid derivatives, considered as key contributors to the antioxidant and hypoglycemic effects of the extracts.

The EtOH and EL extracts exhibited no significant toxicity when administered to the human colon fibroblast cell line and were effective in reducing vanadium toxicity when combined with BMOV or BPOV. Notably, EL extracts demonstrated significant activity in decreasing the toxicity of BMOV across a wide concentration range (80–319 µg/ml). Moreover, in vitro assays conducted on adipocytes indicated that BPOV alone enhanced glucose uptake more effectively than insulin, and this effect was further intensified when BPOV was combined with either EtOH or EL extracts. This suggests a potential synergistic or additive hypoglycemic effect that could be beneficial for managing blood glucose levels in diabetic conditions.

The hypoglycemic action of the most promising formulations was confirmed by immunocytochemical experiments which demonstrated that their mechanism of action may involve the translocation of the GLUT4 transporter to the cell membrane in adipocytes, a pathway associated with enhanced glucose uptake. This evidence suggests that these formulations mimic the insulin-signaling pathway, making them promising candidates for further investigation in diabetes treatment.

In summary, the findings emphasize the advantages of integrating olive leaf extracts with vanadium complexes to enhance antidiabetic effectiveness while minimizing toxicity. This combination enables the administration of lower doses of metal compounds necessary for achieving the desired pharmacological effects, thereby supporting the continued development and refinement of these formulations for clinical use.

## Supplementary Information

Below is the link to the electronic supplementary material.

Supplementary file1 (DOCX 222 KB)

## Data Availability

Data is provided within the manuscript or supplementary information files.

## Footnotes

1 International Diabetes Federation, Facts & figures. Retrieved Accessed on January 16, 2024, https://idf.org/about-diabetes/diabetes-facts-figures/

2 CFM Pharma, Retrieved Accessed on January 16, 2024, https://cfmpharma.com/the-new-drug-vanadis/, https://cfmpharma.com/development-program-of-vanadis/.

3 Medesis Pharma, Retrieved Accessed on January 16, 2024, https://www.medesispharma.com/non-classe/drug-to-treat-type-2-diabetes/

4 Commission Regulation (EC), No 1170/2009 of 30 November 2009 amending Directive 2002/46/EC of the European Parliament and of Council and Regulation (EC) No 1925/2006 of the European Parliament and of the Council as regards the lists of vitamin and minerals and their forms that can be added to foods, including food supplements http://data.europa.eu/eli/reg/2009/1170/oj.
