# Supplementary material for: Antidiabetic potential of vanadium complexes combined with olive leaf extracts: a viable approach to reduce metal toxicity
Source: Biometals. 2025 Feb 27;38(2):683–98. doi: 10.1007/s10534-025-00673-x (PMC11965145; doi:10.1007/s10534-025-00673-x)
Supplement: Supplementary file 1 — Supplementary file1 (DOCX 222 KB) [file 10534_2025_673_MOESM1_ESM.docx]

**SUPPLEMENTARY MATERIAL**

**Antidiabetic potential of vanadium complexes combined with olive leaf extracts: a viable approach to reduce metal toxicity**

Daniele Sanna^† 1^, Angela Fadda^†2^, Milena Casula^†3^, Grazia Palomba^3^, Maria Cristina Sini^3^, Maria Colombino^3^, Carla Rozzo^3^, Giuseppe Palmieri^4^, Carmela Gallo^5^, Dalila Carbone^5^, Laura Siracusa^6^, Luana Pulvirenti^6^, Valeria Ugone*^1^

^1^ Consiglio Nazionale delle Ricerche, Istituto di Chimica Biomolecolare, Traversa La Crucca 3, 07100 Sassari, Italy

[*valeria.ugone@cnr.it*](mailto:valeria.ugone@cnr.it)

^2^Consiglio Nazionale delle Ricerche, Istituto di Scienze delle Produzioni Alimentari, Traversa La Crucca 3, 07100 Sassari, Italy

^3^Consiglio Nazionale delle Ricerche, Istituto di Ricerca Genetica e Biomedica, Traversa La Crucca 3, 07100 Sassari, Italy.

^4^Dipartimento di Medicina, Chirurgia e Farmacia, Università di Sassari, Viale San Pietro 43, 07100 Sassari, Italy

^5^ Consiglio Nazionale delle Ricerche, Istituto di Chimica Biomolecolare, Via Campi Flegrei, 34, 80078 Pozzuoli NA, Italy

^6^ Consiglio Nazionale delle Ricerche, Istituto di Chimica Biomolecolare, Via Paolo Gaifami, 18, 95126 Catania, Italy

^† These authors contributed equally to this work and share first authorship.^


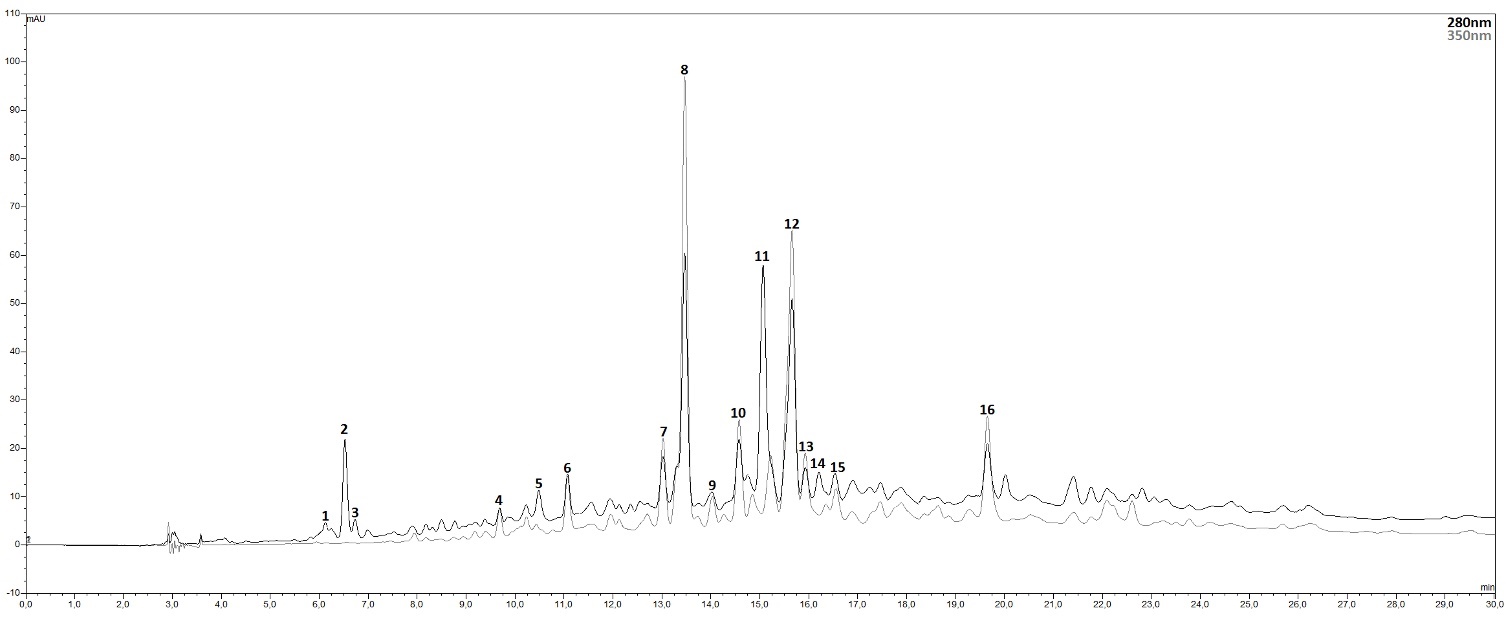


**Fig. S1** HPLC/Uv-vis-DAD chromatogram, visualized at 280 nm (black line) and 350 nm (grey line) of the ethyl lactate extract from olive leaves object of this study. Numbers refer to Table 2; see text for further details.


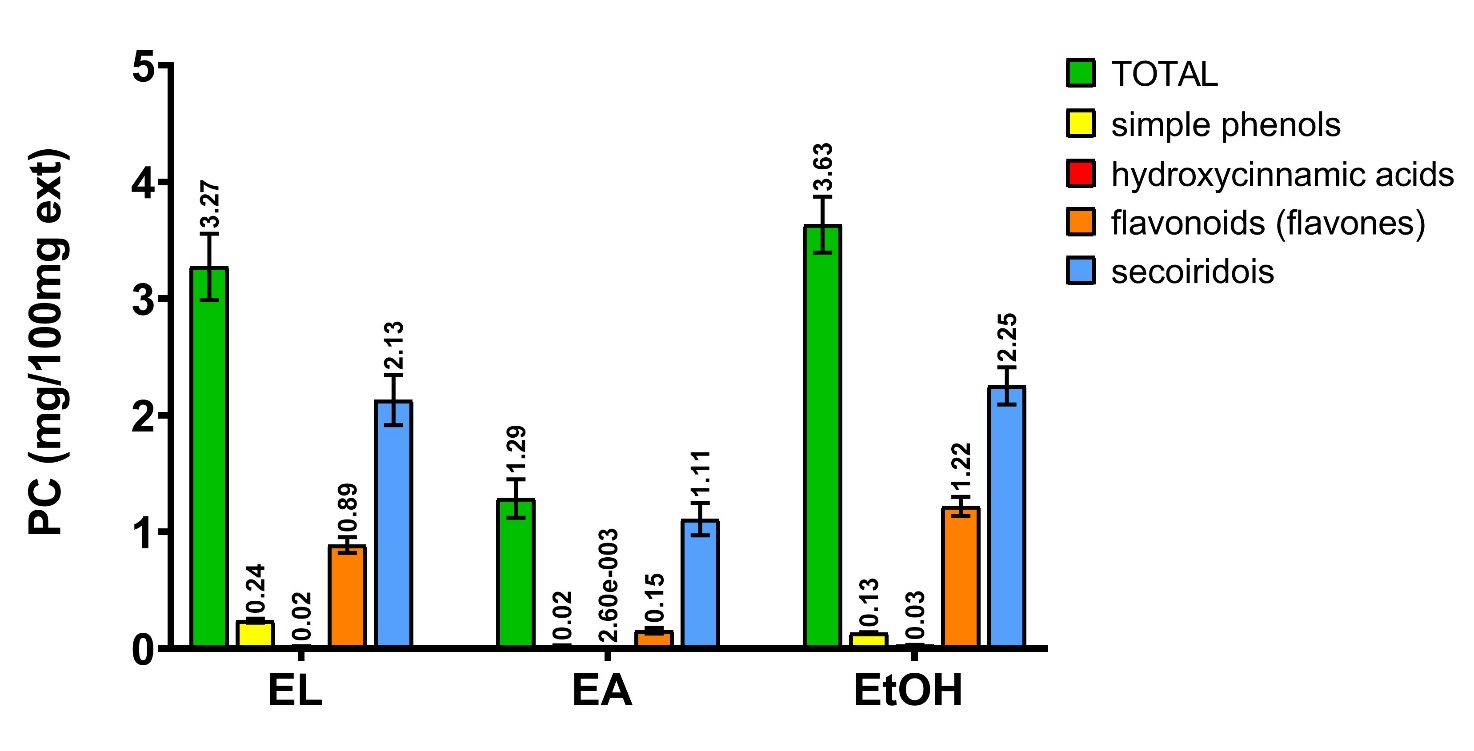


**Fig. S2** Yield in polyphenols (mg/100 mg), divided into biochemical classes, of olive leaves extracts obtained with different solvents. EL = ethyl lactate; EA = ethyl acetate; EtOH = ethanol; results are reported as mean of three replicates. See material and methods and text for further details.
